# The relationship between serum klotho and cognitive performance in a nationally representative sample of US adults

**DOI:** 10.3389/fnagi.2023.1053390

**Published:** 2023-02-02

**Authors:** Deng Linghui, Yu Simin, Zhang Zilong, Li Yuxiao, Qiu Shi, Dong Birong

**Affiliations:** ^1^National Clinical Research Center of Geriatrics, The Center of Gerontology and Geriatrics, West China Hospital, Sichuan University, Chengdu, China; ^2^Department of Urology, Institute of Urology and National Clinical Research Center for Geriatrics, West China Hospital of Sichuan University, Chengdu, China

**Keywords:** cognitive performance, klotho, aging, NHANES, older adults

## Abstract

**Purpose:**

Aging is the primary risk factor for cognitive decline. Serum klotho, as an anti-aging protein, may be involved in cognitive decline. Thus, we aim to explorer the correlation between serum klotho and cognitive performance among an older adult population in the United States.

**Methods:**

We performed a cross-sectional study using data from NHANES 2011–2014. Serum klotho was analyzed by ELISA. Cognitive function was measured by Establish a Registry for Alzheimer’s Disease (CERAD) test, Animal Fluency test and Digit Symbol Substitution Test (DSST) score. The relationship between serum klotho and cognition was analyzed by a multivariable regression model.

**Results:**

A total of 2,171 participants aged 60–79 years were included. Median serum klotho concentration was 851.52 pg./ml (SD = 294.07). We also categorized serum klotho concentrates into quartiles. After fully adjusting pertinent variables, compared to those with lowest klotho levels (206.3–658.4 pg./ml), individuals with highest klotho concentrates (983.3–3,456 pg./ml) had a higher CERAD score [β (95%CI): 0.97 (0.25, 1.69) *p* = 0.008] and DSST score [β (95%CI): 1.86 (0.25, 3.47), *p* = 0.024].

**Conclusion:**

Our findings indicated that, among the general population of American older adults, serum klotho concentrates may serve as a marker of cognitive health. The benefits of klotho on aging process and neurodegenerative disorders should be paid more attention.

## Introduction

The world’s population is aging rapidly, and cognitive impairment is a common symptom of ageing and neurodegenerative disorders (Minhas et al., 2021). Alzheimer’s disease and other dementias was currently ranked as the seventh leading cause of death globally, and are projected to affect 115.4 million people by 2050 (World Health Organization, 2012; Alzheimer’s Association. Alzheimer’s Facts and Figures, 2021). Strategies to preserve cognitive health, which include the ability to clearly think, learn, and remember, would impact the quality of life for a significant proportion of the ageing population.

Among ageing biomarkers, klotho gene, named after the Goddess in Greek mythology who spun the thread of life, was identified as an aging suppressor gene for the first time in 1997 (Kuro-o et al., 1997; Kuro, 2019). Klotho knockout mice manifest a syndrome resembling premature human aging with hyperphosphatemia, osteopenia, atrophy of multiple organs, atherosclerosis, vascular and soft tissue calcification, with a resultant consequence of shorten life span (Kuro-o et al., 1997; Kawaguchi et al., 1999; Hu et al., 2011; Kuro, 2019). Furthermore, administration of exogenous klotho in Klotho-deficient mice extends lifespan (Chen et al., 2013). Overexpression of the klotho gene can increase longevity (Kurosu et al., 2005; Dubal et al., 2015).

Studies have found that normal brain aging in rhesus monkeys was associated with down-regulation of klotho expression, and loss of klotho could accelerate the development of cognitive deficits (Abraham et al., 2016). Animal experiments had further proved that klotho transgenic mice were stronger than the wild type in spatial learning and memory function, working and instrumental memory ability, and had nothing to do with the age of the mice. Kuang et al. also found that AD mice overexpressing klotho achieved better results in a variety of learning and memory tests (Kuang et al., 2014). Although evidence that klotho expression is substantially linked to aging and lifespan in model animals, research in human populations is limited. Clinical studies have shown that klotho protein levels in the cerebrospinal fluid of patients are significantly reduced compared to elderly patients without AD (Semba et al., 2014). Paroni et al. reported that increasing the level of klotho protein can improve cognitive function in early AD patients and delay the progression of the disease (Paroni et al., 2019).

Nevertheless, it is presently uncertain if prior findings can be extrapolated to the broader adult population. We investigate whether blood klotho concentrations are linked with cognitive impairment in a nationally representative sample of people in the United States.

## Materials and methods

### Data sources

The National Health and Nutrition Examination Survey (NHANES) is an ongoing health program of the civilian non-institutionalized American resident population. The data from NHANES is available on the website.^1^ To find participants who are representative of the target population, it adopts a complicated, stratified, multi-stage probability sampling procedure.

Data from two continuous NHANES cycles were merged for this analysis: 2011–2012 and 2013–2014. During this time, 19,931 people aged 60 to 79 years old who agreed to have their excess serum utilized for future study were assessed. As a result, we limited our analysis to participates with available serum klotho concentrations and cognitive assessments information (*n* = 2,171).

### Assessment of klotho

Serum specimens were collected during 2011–2014. Klotho analyses was performed by a commercially available ELISA kit (IBL, Takasaki, Japan; Kresovich and Bulka, 2022). The assay sensitivity was 6 pg./ml. All samples were examined in duplicate with the average of the two concentrations used as the final value. Each plate also contained two quality control samples analyzed in duplicate. In this study, we categorized serum klotho concentrates(pg/ml) into quartiles (Q1: <25th percentile, 206.3–658.4 pg./ml, Q2: 25th–50th percentile 658.5–809.2 pg./ml, Q3: 50th–75th percentile 809.3–982.9 pg./ml, Q4: ≥75th percentile 983.3–3,456 pg./ml), and Q1 was regarded as the reference category. The continuous Klotho was lg-transformed due to its high-skew distribution.

### Assessment of cognition

Cognitive evaluation in the study was measured by using three different tests representing different domains of cognition among participants aged 60 years or older. The Digit Symbol Substitution Test (DSST) measured performance on speed tests (Jaeger, 2018). The Consortium to Establish a Registry for Alzheimer’s Disease (CERAD) measured immediate and delayed memory (Kivipelto et al., 2013). The Animal Fluency test (AFT) asked participants to name as many animals as possible within 1 min and measured verbal fluency (Chen et al., 2017). Currently, the CERAD, AFT and DSST tests do not have a gold standard of cutoff point for to determining low cognitive performance. Thus, consistent with the methods used in the published literature, we used the 25^th^ percentile of the scores as the cutoff points (Chen et al., 2017).

### Covariates

Investigation covariates incorporated three parts: demographic variables, anthropometric variables and lifestyle variables. Gender, age, education level, and race/ethnicity were all considered demographic factors. For socioeconomic status, the poverty-income ratio (PIR) was adopted, and marital status was categorized as living alone and live with partners. Other information, such as cigarettes smoking, alcohol consumption, and physical activity, were also included as possible confounder. Three levels of smoking status was defined as never (less than 100 cigarettes), former (no less than 100 cigarettes without current smoking), or current (no less than 100 cigarettes with current smoking). Alcohol drinking status was classified into none (0 g/day), moderate (0.1–27.9 g/day for men and 0.1–13.9 g/day for women), heavy (28.0 g or more/day for man and 14.0 g or more/day for women). A categorical variable was defined to assess the physical activity intensity: less than moderate as (0 min/week of moderate-to-vigorous physical activity), moderate as between 0 and 150 min/week, and vigorous as more than 150 min/week according to the national physical activity guidelines. Additionally, we generated a composite cardiovascular disease (CAD) score that aggregated multiple risk factors. One score was assigned to each of the three current risk factors: history of coronary heart disease, hypertension, or stroke, and two points were added for diabetes. Scores ranged from zero to five (Golub et al., 2020).

### Statistical methods

Considering the complex study design of NHANES and in order to make the study population nationally representative, a sample weight was put on each participant. In accordance with the NHANES Survey Methods and Analytic Guidelines, recommended weighting methodology was applied during the analysis process.^2^ Results with *p* < 0.05 were regarded as significant. Klotho concentrations were log transformed due to its skewed distribution. Multivariable linear regression was performed to assess the association between Klotho and CERAD, AFT, and DSST scores, and multivariable logistic regression was used to assess the association between Klotho and log cognitive performance defined with the cutpoints of 25^th^ percentile in CERAD, AFT, or DSST scores. Following the Strengthening the Reporting of Observational Studies in Epidemiology (STROBE) statement guideline (von Elm et al., 2014), we constructed three models: Non-adjusted, no covariates were adjusted; Model 1, gender, age, race, PIR, education level, and marital status were adjusted; Model 2, including variables from model 1 with smoking, alcohol intake, CAD score and BMI added. We also converted the serum klotho concentrates into a categorical variable by quartile and calculated the P for trend. Furthermore, the subgroup analyses were performed to investigate the interaction between each covariate in Model 2 and Klotho quartiles on cognitive scores, adjusting for the remaining covariates. Age was categorized into younger and older group with the cutpoint of 67 years, the median of the age among all selected participants.

## Results

### Sample characteristics

The characteristics information were presented in Table 1. 2,171 eligible participants with a mean age of 67.58 years (ranged from 60 to 79 years) were involved in the current study, and approximately half are female (51.45%). The weighted study population was primarily non-Hispanic white (42.75%), receiving education above high school (51.81%), married or living with partner (61.20%), having none alcohol intake (79.32%), having never smoked (48.80%) and doing less than moderate physical activity (56.38%). Median serum klotho concentration was 851.52 pg./ml (SD = 294.07). Compared with individuals with normal cognitive performance, those who reported low cognitive performance were more likely to be older, male, nondrinkers, thinner, have less educational levels, lower PIR, more comorbidities, live alone and do less physical activities (Table 1).

**Table 1:** Characteristics of the study population.

	Total	Klotho group				
		Q1	Q2	Q3	Q4	*P* value
N		1654	484			
Age	67.58 (5.46)	68.38 (5.55)	67.64 (5.46)	67.18 (5.36)	67.12 (5.37)	<0.001
Klotho (pg/ml)	851.52 (294.07)	206.30–658.40	658.50–809.20	809.30–982.90	983.30–3456.00	
Gender						0.018
Male	1054 (48.55%)	268 (49.36%)	286 (52.77%)	265 (48.80%)	235 (43.28%)	
Female	1117 (51.45%)	275 (50.64%)	256 (47.23%)	278 (51.20%)	308 (56.72%)	
Race						0.001
Mexican American	230 (10.59%)	53 (9.76%)	55 (10.15%)	63 (11.60%)	59 (10.87%)	
Other Hispanic	249 (11.47%)	45 (8.29%)	61 (11.25%)	73 (13.44%)	70 (12.89%)	
Non-Hispanic White	928 (42.75%)	254 (46.78%)	248 (45.76%)	233 (42.91%)	193 (35.54%)	
Non-Hispanic Black	529 (24.37%)	137 (25.23%)	122 (22.51%)	108 (19.89%)	162 (29.83%)	
Other Race	235 (10.82%)	54 (9.94%)	56 (10.33%)	66 (12.15%)	59 (10.87%)	
PIR						0.079
<1.3	608 (30.49%)	165 (30.39%)	150 (27.68%)	159 (29.28%)	134 (24.68%)	
1.3-3.5	733 (36.76%)	218 (40.15%)	243 (44.83%)	205 (37.75%)	244 (44.94%)	
>3.5	653 (32.75%)	160 (29.47%)	149 (27.49%)	179 (32.97%)	165 (30.39%)	
Education level						0.338
Less than high school	535 (25.11%)	136 (25.05%)	151 (27.86%)	127 (23.39%)	121 (22.28%)	
High school or GED	492 (23.09%)	126 (23.20%)	119 (21.96%)	115 (21.18%)	132 (24.31%)	
Above high school	1104 (51.81%)	281 (51.75%)	272 (50.18%)	301 (55.43%)	290 (53.41%)	
marital						0.619
Married or living with partner	1328 (61.20%)	345 (63.54%)	331 (61.07%)	327 (60.22%)	326 (60.04%)	
Living alone	842 (38.80%)	198 (36.46%)	211 (38.93%)	216 (39.78%)	217 (39.96%)	
BMI groups						0.790
<=25	532 (24.74%)	125 (23.02%)	130 (23.99%)	135 (24.86%)	142 (26.15%)	
25-30	772 (35.91%)	192 (35.36%)	204 (37.64%)	186 (34.25%)	190 (34.99%)	
>30	846 (39.35%)	226 (41.62%)	208 (38.38%)	222 (40.88%)	211 (38.86%)	
CAD score						0.008
0	607 (29.44%)	118 (21.73%)	164 (30.26%)	163 (30.02%)	162 (29.83%)	
1	725 (35.16%)	193 (35.54%)	162 (29.89%)	183 (33.70%)	187 (34.44%)	
2	730 (35.40%)	232 (42.73%)	216 (39.85%)	197 (36.28%)	194 (35.73%)	
Smoking status						0.009
Never	1059 (48.80%)	254 (46.78%)	233 (42.99%)	278 (51.20%)	295 (54.33%)	
Former	851 (39.22%)	223 (41.07%)	240 (44.28%)	204 (37.57%)	184 (33.89%)	
Current	260 (11.98%)	66 (12.15%)	69 (12.73%)	61 (11.23%)	64 (11.79%)	
Alcohol intake						0.021
None	1596 (79.32%)	432 (79.56%)	419 (77.31%)	450 (82.87%)	454 (83.61%)	
Moderate	175 (8.70%)	41 (7.55%)	46 (8.49%)	42 (7.73%)	46 (8.47%)	
Heavy	241 (11.98%)	70 (12.89%)	77 (14.21%)	51 (9.39%)	43 (7.92%)	
Physical activity						0.603
Less than moderate	1224 (56.38%)	325 (59.85%)	299 (55.17%)	293 (53.96%)	307 (56.54%)	
Moderate	188 (8.66%)	45 (8.29%)	46 (8.49%)	49 (9.02%)	48 (8.84%)	
Vigorous	759 (34.96%)	173 (31.86%)	197 (36.35%)	201 (37.02%)	188 (34.62%)	

CERAD, The Consortium to Establish a Registry for Alzheimer’s Disease; AFT, Animal Fluency test; DSST, The Digit Symbol Substitution Test; PIR, ratio of family income to poverty; GED, general educational development; BMI, body mass index.

### Multivariable regression analysis of klotho with cognitive function

**Figure 1:**
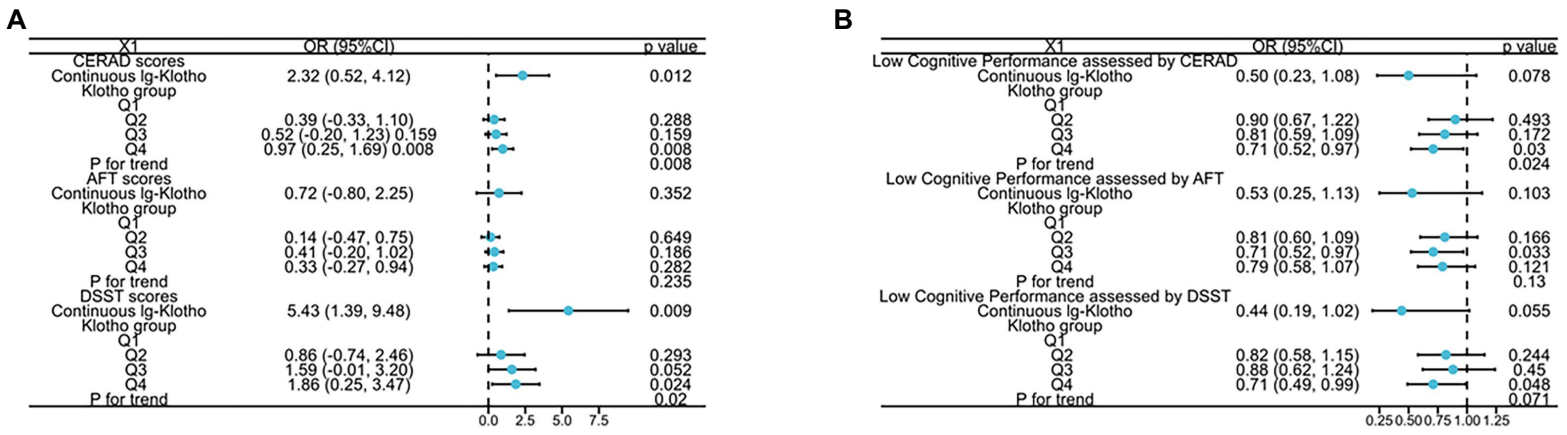
The relationship between **(A)** both lg-transformed and categorical Klotho and CERAD, AFT, and DSST scores; **(B)** both lg-transformed and categorical Klotho and low cognitive performance assessed by CERAD, AFT, and DSST scores.

The relationship between serum klotho concentrates and different dimensions of cognitive performance (assessed by DSST, CERAD, AFT scores) among the US older adults was demonstrated in Figure 1 and Supplementary Table 1. In the Non-adjusted Model, the analysis revealed a substantial positive association between klotho concentrates and cognitive performance measured by CERAD and DSST scores (log-transformed klotho: *β* = 3.19, 95%CI 1.22 to 5.16, *p* = 0.001 for CERAD score, *β* = 7.28, 95%CI 1.93 to 12.62, *p* = 0.008 for DSST score; categorical klotho: p for trend all<0.05). In the Model II, compared to those with lowest klotho levels (206.3–658.4 pg./ml), individuals with highest klotho concentrates (983.3–3,456 pg./ml) had a higher CERAD score [0.97 (0.25, 1.69) *p* = 0.008] and DSST score [1.86 (0.25, 3.47), *p* = 0.024]. A positive association between klotho concentrates and cognitive performance measured by CERAD and DSST scores was shown in Model II (log-transformed klotho: *β* = 2.32, 95%CI 0.52 to 4.12, *p* = 0.012 for CERAD score, *β* = 5.43, 95%CI 1.39 to 9.48, *p* = 0.009 for DSST score; categorical klotho: p for trend all<0.05).

Additionally, to further detect the correlation of klotho concentrates with cognitive impairment, we stratified the dimensions of cognitive performance into categorical variable by the 25th percentiles, a cutoff point for lower cognitive performance, and a similar trend was discovered. Compared to participates with Q1 group of klotho levels, the risk of lower cognitive performance significantly decreased 29% in Q4 group, assessed by DSST and CERAD tests. Moreover, p for trend is 0.024 assessed by CERAD test.

### Subgroup analysis

In subgroup analysis of the relationship between klotho levels and DSST scores (shown in Supplementary Table 2), after adjusting for potential confounders, the interaction test was not statistically significant for gender, age, marital status, PIR, educational level, BMI, alcohol intake, smoking status, CAD score, and physical activity (*P* for interaction >0.05). Therefore, we do not detect any potential evidence that there are systematic differences in relationship in different subgroups, suggesting that our main results remain stable.

## Discussion

To our knowledge, this work is the first research on the relationship between serum klotho levels and cognitive impairment among the general older adults in the United States. A positive association between cognitive function and klotho level was found. The observed relationship was robust and independent of potential confounders such as gender, age, race, education level, marital status, ratio of family income to poverty, alcohol intake, smoking, CAD score and BMI.

The klotho protein may protect against cognitive impairment *via* multiple biological mechanisms. In transgenic mice that systemic overexpress klotho, the mice outperformed controls in memory and spatial learning (Dubal et al., 2014). In model animals, elevating klotho enhanced long-term potentiation, a kind of synaptic plasticity, and enriched the N-methyl-D-aspartate receptor (NMDAR) subunit GluN2B, which is critical for memory and learning (Dubal et al., 2014). Elevated Klotho also promoted the expression of FOS by activating NMDA receptor, which is beneficial for memory consolidation (Dubal et al., 2014). In wildtype mice, long-lasting enhancement of memory and learning capabilities are noticed after a single adenovirus injection of secreted klotho into the CNS (Masso et al., 2018). Viral expression of secreted human klotho in the hippocampus region altered memory formation and social behavior, such as improving object recognition/location, passive avoidance memory and increasing hippocampal synaptic transmission (Li et al., 2019).

Oxidative stress has long been associated with ageing-related cognitive deficits (Harman, 1981). During ageing, oxidative damage to the synapse in the hippocampus and cerebral cortex leads to cognitive impairment (Fukui et al., 2002). Overall oxidative stress burden in the brain is generally increased in klotho knockout mice (Kurosu et al., 2005), suggesting that klotho plays an antioxidant role in the CNS. In addition, upregulation of the klotho ameliorate ageing-related memory impairment and decreased oxidative damage in senescence-accelerated model mice (Zhou et al., 2018). Soluble klotho was reported to exert protective effects on neurons against oxidative damage induced by glutamate and amyloid-β (Zeldich et al., 2014; Sedighi et al., 2021). Furthermore, klotho reduced multiple pro-neuroinflammatory cytokines (IL-1β, TNF-α) in cells exposed to amyloid-β (Sedighi et al., 2021).

In accordance with previous studies, our research indicated that serum klotho concentrate may be a potential biomarker of neurodegeneration and cognitive health. A pilot case control study recruiting 280 older adults reported that klotho concentrations were significant correlated with higher scores in cognitive function tests, including the word fluency tests and Mini-Mental State Examination (MMSE) (Kuriyama et al., 2018). A prospective cohort study including Italian adults aged 55 or older also showed that lower plasma klotho levels were linked with elevated risk of meaningful decline in MMSE (Shardell et al., 2016). Moreover, klotho concentrates both in CSF and serum are positively related with MMSE scores despite of apoE4 status or gender (Kundu et al., 2022). A longitudinal study including 527 men aged 71–87 years by Almeida et al. reported that Klotho variant is associated with an increase in the incidence of dementia in older men (Almeida et al., 2017).

There are some limitations. Firstly, in the NHANES database, only individuals aged 60–79 years old had available data of both klotho concentrates and cognitive assessments, so extrapolation of trend on youngers remains unknown. Secondly, although a number of latent confounders have been adjusted including demographic characteristics, self-reported lifestyle factors and health status, the probability of unmeasured and remaining confounders cannot be completely eliminate. Thirdly, due to the design of this observational study, causal relationship cannot be established. However, we used a nationally representative sample of order adults in the United States with a large sample size, which improved methodological issues and contributed to the robustness of our findings. Fourthly, Klotho values were only measured in serum samples. The values in cerebrospinal fluid were not measured, which might be more relevant to cognitive function. However, in previous studies, also circulating Klotho was reported to be higher in cerebrospinal fluid than serum, high corelationship between Klotho levels in serum and in cerebrospinal fluid has been demonstrated (Gaitán et al., 2022; Kundu et al., 2022). Further prospective studies with higher quality are needed to focus on klotho levels in younger adults, and explore whether the influence of klotho on domain-specific cognition changes.

## Conclusion

In conclusion, our findings indicate that serum klotho concentrates were related with cognitive performance among the US older adults. More high-quality prospective studies are needed to further explorer the effects of klotho levels on domain-specific cognitive function.

## Data availability statement

The original contributions presented in the study are included in the article/Supplementary material, further inquiries can be directed to the corresponding authors.

## Ethics statement

The studies involving human participants were reviewed and approved by the NCHS Ethics Review Board, and written informed consents were signed by all participants. The patients/participants provided their written informed consent to participate in this study.

## Author contributions

DB had access to all the data in the study and takes responsibility for the integrity of the data and the accuracy of the data analysis. DB, DL, QS, and YS designed the study. ZZ, DL, and QS acquired and analyzed the data. DL and YS drafted the manuscript. ZZ and LY supervised all aspects of the study. DB and QS revised the manuscript critically for important intellectual content. All authors contributed to the article and approved the final version.

## Funding

This work was supported by Programs from Science and Technology Department of Sichuan Province (2021YJ0462), Project of Max Cynader Academy of Brain Workstation, WCHSCU (HXYS19005), China Postdoctoral Science Foundation (2020M670057ZX), Post-Doctor Research Project, West China Hospital, Sichuan University (2019HXBH092) and the Health and Family Planning Commission of Sichuan Province (20PJ039).

## Conflict of interest

The authors declare that the research was conducted in the absence of any commercial or financial relationships that could be construed as a potential conflict of interest.

## Publisher’s note

All claims expressed in this article are solely those of the authors and do not necessarily represent those of their affiliated organizations, or those of the publisher, the editors and the reviewers. Any product that may be evaluated in this article, or claim that may be made by its manufacturer, is not guaranteed or endorsed by the publisher.

## Footnotes

^1^ https://www.cdc.gov/nchs/data_access/index.htm

^2^ https://wwwn.cdc.gov/nchs/nhanes/tutorials/weighting.aspx
