# Supplementary material for: The relationship between serum klotho and cognitive performance in a nationally representative sample of US adults
Source: Front Aging Neurosci. 2023 Feb 2;15:1053390. doi: 10.3389/fnagi.2023.1053390 (PMC9932504; doi:10.3389/fnagi.2023.1053390)
Supplement: Supplementary file 1 [file Data_Sheet_1.docx]

Supplementary Table 1: The relationship between klotho concentration and cognitive assessments.

| Exposure | Non-adjusted | Model I | Model II |
| --- | --- | --- | --- |
| CERAD scores | | | |
| Continuous lg-Klotho | 3.19 (1.22, 5.16) p=0.001 | 2.35 (0.55, 4.15) p=0.011 | 2.32 (0.52, 4.12) p=0.012 |
| Klotho group |  |  |  |
| Q1 | 0 (Ref.) | 0 (Ref.) | 0 (Ref.) |
| Q2 | 0.43 (-0.36, 1.22) 0.284 | 0.47 (-0.26, 1.17) 0.212 | 0.39 (-0.33, 1.10) 0.288 |
| Q3 | 0.89 (0.09, 1.68) 0.027 | 0.55 (-0.17, 1.26) 0.137 | 0.52 (-0.20, 1.23) 0.159 |
| Q4 | 1.31 (0.52, 2.09) 0.001 | 0.97 (0.25, 1.69) 0.008 | 0.97 (0.25, 1.69) 0.008 |
| P for trend | ＜0.001 | 0.010 | 0.008 |
| AFT scores | | | |
| Continuous lg-Klotho | 0.83 (-0.86, 2.53) p=0.336 | 0.89 (-0.64, 2.43) p=0.252 | 0.72 (-0.80, 2.25) p=0.352 |
| Klotho group |  |  |  |
| Q1 | 0 (Ref.) | 0 (Ref.) | 0 (Ref.) |
| Q2 | 0.32 (-0.36, 1.00) 0.353 | 0.26 (-0.36, 0.87) 0.412 | 0.14 (-0.47, 0.75) 0.649 |
| Q3 | 0.78 (0.09, 1.46) 0.026 | 0.49 (-0.12, 1.10) 0.118 | 0.41 (-0.20, 1.02) 0.186 |
| Q4 | 0.36 (-0.32, 1.04) 0.302 | 0.39 (-0.23, 0.99) 0.218 | 0.33 (-0.27, 0.94) 0.282 |
| P for trend | 0.256 | 0.204 | 0.235 |
| DSST scores | | | |
| Continuous lg-Klotho | 7.28 (1.93, 12.62) p=0.008 | 5.80 (1.70, 9.89) p=0.006 | 5.43 (1.39, 9.48) p=0.009 |
| Klotho group |  |  |  |
| Q1 | 0 (Ref.) | 0 (Ref.) | 0 (Ref.) |
| Q2 | 1.36 (-0.78, 3.50) 0.212 | 1.18 (-0.45, 2.81) 0.155 | 0.86 (-0.74, 2.46) 0.293 |
| Q3 | 3.04 (0.91, 5.18) 0.005 | 1.81 (0.18, 3.44) 0.030 | 1.59 (-0.01, 3.20) 0.052 |
| Q4 | 2.43 (0.29, 4.56) 0.026 | 1.94 (0.30, 3.57) 0.020 | 1.86 (0.25, 3.47) 0.024 |
| P for trend | 0.018 | 0.021 | 0.020 |
|  |  |  |  |
| Low Cognitive Performance assessed by CERAD | | | |
| Continuous lg-Klotho | 0.43 (0.21, 0.89) p=0.022 | 0.53 (0.25, 1.13) p=0.098 | 0.50 (0.23, 1.08) 0.078 |
| Klotho group |  |  |  |
| Q1 | 1.00 (Ref.) | 1.00 (Ref.) | 1.00 (Ref.) |
| Q2 | 0.88 (0.67, 1.16) 0.378 | 0.86 (0.64, 1.16) 0.317 | 0.90 (0.67, 1.22) 0.493 |
| Q3 | 0.76 (0.57, 1.01) 0.054 | 0.79 (0.59, 1.08) 0.140 | 0.81 (0.59, 1.09) 0.172 |
| Q4 | 0.68 (0.51, 0.90) 0.008 | 0.72 (0.53, 0.96) 0.040 | 0.71 (0.52, 0.97) 0.030 |
| P for trend | 0.005 | 0.038 | 0.024 |
| Low Cognitive Performance assessed by AFT | | | |
| Continuous lg-Klotho | 0.52 (0.25, 1.06) p=0.070 | 0.51 (0.24, 1.09) p=0.083 | 0.53 (0.25, 1.13) p=0.103 |
| Klotho group |  |  |  |
| Q1 | 1.00 (Ref.) | 1.00 (Ref.) | 1.00 (Ref.) |
| Q2 | 0.79 (0.59, 1.04) 0.092 | 0.79 (0.58, 1.06) 0.114 | 0.81 (0.60, 1.09) 0.166 |
| Q3 | 0.66 (0.49, 0.88) 0.004 | 0.70 (0.52, 0.96) 0.025 | 0.71 (0.52, 0.97) 0.033 |
| Q4 | 0.79 (0.60, 1.05) 0.103 | 0.78 (0.58, 1.05) 0.105 | 0.79 (0.58, 1.07) 0.121 |
| P for trend | 0.101 | 0.122 | 0.130 |
| Low Cognitive Performance assessed by DSST | | | |
| Continuous lg-Klotho | 0.50 (0.25, 1.02) p=0.056 | 0.45 (0.20, 1.02) p=0.056 | 0.44 (0.19, 1.02) p=0.055 |
| Klotho group |  |  |  |
| Q1 | 1.00 (Ref.) | 1.00 (Ref.) | 1.00 (Ref.) |
| Q2 | 0.82 (0.62, 1.09) 0.170 | 0.78 (0.56, 1.10) 0.156 | 0.82 (0.58, 1.15) 0.244 |
| Q3 | 0.82 (0.62, 1.09) 0.177 | 0.85 (0.60, 1.20) 0.352 | 0.88 (0.62, 1.24) 0.450 |
| Q4 | 0.76 (0.57, 1.01) 0.063 | 0.72 (0.51, 1.01) 0.054 | 0.71 (0.49, 0.99) 0.048 |
| P for trend | 0.085 | 0.090 | 0.071 |

CERAD, The Consortium to Establish a Registry for Alzheimer’s Disease; AFT, Animal Fluency test; DSST, The Digit Symbol Substitution Test.

Non-adjusted, no covariates were adjusted;

Model 1, gender, age, race, PIR, education level, and marital status were adjusted;

Model 2, gender, age, race, PIR, education level, marital status, smoking, alcohol intake, CAD score and BMI were adjusted.

Supplementary Table 2: Subgroup analysis of the relationship between klotho concentrates (quartiles) and DSST scores.

| Klotho Quartiles |  | DSST scores |  |  |
| --- | --- | --- | --- | --- |
| Age | Younger | Older | P for interaction |  |
| N | 1025 | 1065 |  |  |
| Q1 | 0 (Ref.) | 0 (Ref.) | 0.275 |  |
| Q2 | 1.54 (-0.67, 3.74) p=0.172 | 3.04 (0.90, 5.17) p=0.005 |  |  |
| Q3 | 3.20 (1.02, 5.38) p=0.004 | 2.77 (0.56, 4.97) p=0.014 |  |  |
| Q4 | 1.47 (-0.80, 3.75) p=0.204 | 4.12 (1.87, 6.37) p＜0.001 |  |  |
| p for trend | 0.168 | ＜0.001 |  |  |
| Gender | Male | Female | P for interaction |  |
| N | 1015 | 1075 |  |  |
| Q1 | 0 (Ref.) | 0 (Ref.) | 0.799 |  |
| Q2 | 1.67 (-0.25, 3.59) 0.088 | 2.65 (0.38, 4.93) 0.022 |  |  |
| Q3 | 1.71 (-0.26, 3.68) 0.089 | 3.65 (1.37, 5.92) 0.002 |  |  |
| Q4 | 1.87 (-0.28, 4.01) 0.088 | 3.13 (0.87, 5.39) 0.007 |  |  |
| p for trend | 0.108 | 0.011 |  |  |
| Marital status | Married or living with partner | Living alone | P for interaction |  |
| N | 1286 | 804 |  |  |
| Q1 | 0 (Ref.) | 0 (Ref.) | 0.237 |  |
| Q2 | 0.20 (-1.79, 2.19) 0.847 | 1.70 (-1.01, 4.42) 0.219 |  |  |
| Q3 | 2.24 (0.24, 4.24) 0.028 | 0.37 (-2.34, 3.07) 0.792 |  |  |
| Q4 | 2.35 (0.34, 4.35) 0.022 | 0.83 (-1.89, 3.56) 0.548 |  |  |
| p for trend | 0.007 | 0.806 |  |  |
| PIA | < =1.3 | 1.3-3.5 | > = 3.5 | P for interaction |
| N | 571 | 875 | 644 |  |
| Q1 | 0 (Ref.) | 0 (Ref.) | 0 (Ref.) | 0.470 |
| Q2 | 1.56 (-1.58 4.69) 0.330 | -0.33 (-2.84, 2.17) 0.795 | 2.33 (-0.51, 5.16) 0.108 |  |
| Q3 | 0.55 (-2.57, 3.67) 0.729 | 1.78 (-0.84, 4.40) 0.184 | 3.12 (0.42, 5.82) 0.024 |  |
| Q4 | 2.74 (-0.52, 5.99) 0.099 | 1.37 (-1.16, 3.906) 0.288 | 2.44 (-0.36, 5.24) 0.088 |  |
| p for trend | 0.150 | 0.155 | 0.111 |  |
| Education level | Less than high school | High school or GED | Above high school | P for interaction |
| N | 492 | 474 | 1124 |  |
| Q1 | 0 (Ref.) | 0 (Ref.) | 0 (Ref.) | 0.937 |
| Q2 | 1.32 (-1.84, 4.49) 0.413 | 2.26 (-1.35, 5.87) 0.220 | 0.24 (-1.95, 2.43) 0.831 |  |
| Q3 | 3.32 (0.04, 6.59) 0.047 | 2.35 (-1.40, 6.11) 0.220 | 0.89 (-1.26, 3.03) 0.417 |  |
| Q4 | 0.96 (-2.37, 4.29) 0.571 | 2.29 (-1.31, 5.88) 0.213 | 1.73 (-0.45, 3.92) 0.120 |  |
| p for trend | 0.477 | 0.287 | 0.093 |  |
| BMI | <=25 | 25-30 | = >30 | P for interaction |
| N | 513 | 747 | 830 |  |
| Q1 | 0 (Ref.) | 0 (Ref.) | 0 (Ref.) | 0.171 |
| Q2 | -2.13 (-5.72, 1.45) 0.244 | 1.89 (-0.74, 4.52) 0.159 | 1.98 (-0.53, 4.49) 0.123 |  |
| Q3 | 0.66 (-2.87, 4.18) 0.715 | 3.38 (0.69, 6.08) 0.014 | 0.69 (-1.79, 3.17) 0.587 |  |
| Q4 | 0.68 (-2.84, 4.20) 0.704 | 3.86 (1.17, 6.55) 0.005 | 1.34 (-1.18, 3.86) 0.299 |  |
| p for trend | 0.364 | 0.004 | 0.495 |  |
| Alcohol intake | none | moderate | heavy |  |
| N | 1679 | 172 | 239 |  |
| Q1 | 0 (Ref.) | 0 (Ref.) | 0 (Ref.) | 0.819 |
| Q2 | 1.23 (-0.59, 3.05) 0.186 | -0.33 (-6.61, 5.94) 0.917 | 0.54 (-3.78, 4.86) 0.807 |  |
| Q3 | 2.17 (0.37, 3.96) 0.018 | 1.40 (-5.13, 7.92) 0.675 | -0.98 (-5.84, 3.87) 0.692 |  |
| Q4 | 2.10 (0.31, 3.89) 0.022 | 2.76 (-3.74, 9.26) 0.406 | 1.08 (-4.01, 6.17) 0.678 |  |
| p for trend | 0.020 | 0.321 | 0.817 |  |
| Smoking status | Never | Former | Current | P for interaction |
| N | 1012 | 831 | 247 |  |
| Q1 | 0 (Ref.) | 0 (Ref.) | 0 (Ref.) | 0.412 |
| Q2 | -0.34 (-2.75, 2.07) 0.782 | 2.66 (0.20, 5.13) 0.035 | -1.14 (-5.80, 3.52) 0.632 |  |
| Q3 | 1.49 (-0.83, 3.79) 0.208 | 1.89 (-0.69, 4.48) 0.151 | 0.95 (-3.83, 5.73) 0.696 |  |
| Q4 | 1.39 (-0.90, 3.67) 0.234 | 2.05 (-0.60, 4.71) 0.130 | 2.94 (-1.74, 7.61) 0.219 |  |
| p for trend | 0.132 | 0.233 | 0.126 |  |
| CAD score | 0 | 1 | 2 | P for interaction |
| N | 595 | 700 | 795 |  |
| Q1 | 0 (Ref.) | 0 (Ref.) | 0 (Ref.) | 0.668 |
| Q2 | 0.87 (-2.42, 4.15) 0.605 | -0.25 (-3.07, 2.57) 0.864 | 1.99 (-0.51, 4.48) 0.119 |  |
| Q3 | 0.68 (-2.62, 3.98) 0.688 | 1.61 (-1.10, 4.32) 0.245 | 2.56 (-0.01, 5.12) 0.051 |  |
| Q4 | 2.00 (-1.31, 5.31) 0.237 | 0.44 (-2.29, 3.17) 0.754 | 3.08 (0.53, 5.63) 0.018 |  |
| p for trend | 0.249 | 0.587 | 0.020 |  |
| Physical activity | Less than moderate | Moderate | Vigorous | P for interaction |
| N | 1168 | 182 | 740 |  |
| Q1 | 0 (Ref.) | 0 (Ref.) | 0 (Ref.) | 0.358 |
| Q2 | 1.62 (-0.57, 3.81) 0.147 | -6.32 (-13.12, 0.48) 0.071 | 0.86 (-1.65, 3.38) 0.501 |  |
| Q3 | 2.62 (0.41, 4.83) 0.020 | -2.33 (-8.77, 4.11) 0.480 | 0.59 (-1.94, 3.12) 0.647 |  |
| Q4 | 2.81 (0.61, 5.02) 0.012 | -1.17 (-7.72, 5.38) 0.727 | 0.33 (-2.22, 2.87) 0.802 |  |
| p for trend | 0.012 | 0.860 | 0.020 |  |

CERAD, The Consortium to Establish a Registry for Alzheimer’s Disease; AFT, Animal Fluency test; DSST, The Digit Symbol Substitution Test; PIR, ratio of family income to poverty; GED, general educational development; BMI, body mass index.

Gender, age, race, PIR, education level, marital status, smoking, alcohol intake, CAD score and BMI were adjusted.
